# Reliability and Validity of General Health Questionnaire-12 in Chinese Dental Healthcare Workers During the COVID-19 Pandemic

**DOI:** 10.3389/fpsyt.2021.792838

**Published:** 2022-01-18

**Authors:** Xiaogang Zhong, Xin Jin, Li Yan, Lu Yang, Huiqing Long, Jing Wang, Haiyang Wang, Yiyun Liu, Juncai Pu, Peng Xie, Ping Ji

**Affiliations:** ^1^Key Laboratory of Psychoseomadsy, Stomatological Hospital of Chongqing Medical University, Chongqing, China; ^2^NHC Key Laboratory of Diagnosis and Treatment on Brain Functional Diseases, The First Affiliated Hospital of Chongqing Medical University, Chongqing, China; ^3^College of Basic Medicine, Chongqing Medical University, Chongqing, China; ^4^School of Public Health and Management, Chongqing Medical University, Chongqing, China; ^5^Chongqing Stomatological Association, Chongqing, China

**Keywords:** general health questionnaire, reliability, validity, dental, healthcare workers

## Abstract

**Background:**

The General Health Questionnaire-12 (GHQ-12) is a widely used instrument to assess mental health status. However, little is known about its applicability in Chinese healthcare workers. This study aimed to evaluate the reliability and validity of the GHQ-12 in Chinese dental healthcare workers.

**Methods:**

Dental healthcare workers participated in the first occupational survey in China conducted by the Chongqing Stomatological Association from February 2021 to March 2021 by filling out GHQ-12. The reliability and validity of GHQ-12 were then tested.

**Results:**

A total of 3,020 valid electronic questionnaires were acquired. The positive detection rate of self-reported mental health status was 23.80% (719/3,020). The Cronbach's α coefficient of the GHQ-12 was 0.892, and the Cronbach's α coefficient was 0.877–0.888 after the deletion of individual items, and the split-half reliability was 0.843. The correlation coefficient between the item-total score ranged from 0.465 to 0.762 (*P*<0.05). The exploratory factor analysis found 2 common factors with a factor load of 0.564–0.818. The confirmatory factor analysis showed that the factor load on the specified items was 0.480–0.790.

**Conclusions:**

The two-factor model of GHQ-12 featured good reliability and validity, which could be used to assess the mental health status of Chinese dental healthcare workers.

## Introduction

Mental health problems are widespread among healthcare workers globally, and they are not optimistic in Chinese healthcare workers. A study from China showed that 48.28% of resident physicians had moderate depressive symptoms (1). Another large-scale epidemiological survey showed that 38% of nurses in public hospitals in China had depressive symptoms (2). There are 1.8 physicians for every 1,000 people in China, which is less than half of other developed countries (3, 4). As a direct result of the shortage of health care workers, the workload is enormous. Meanwhile, they also face great academic pressure to achieve promotion in professional titles, all of which have been shown to be risk factors for mental health status (5, 6). With the outbreak of Corona Virus Disease 2019 (COVID-19), the workload of healthcare workers has dramatically increased, and the mental health status of healthcare workers is facing serious challenges.

Medical healthcare workers are high-risk groups for the infection of COVID-19. Especially for dental healthcare workers, as they regularly have face-to-face contact with patients' saliva, blood and crevicular gingival fluid (7, 8). It has been showed that fomites and airborne are the main transmission routes of COVID-19, while previous studies also showed that aerosols might be a medium of transmission. While using instruments in dental procedures, a large number of aerosols will be generated. Virus-laden aerosols, which can hang in the air for a long time (9), are considered to be potentially dangerous particles that can be inhaled into the lungs to spread disease and aggravate the severity of disease progression (10, 11). With the outbreak of COVID-19, dental healthcare workers are continuously exposed to an infectious environment. The huge workload and high-risk working environment put them under great pressure. Therefore, it is necessary to screen the mental health status of dental healthcare workers efficiently.

Currently, there are many scales for the self-measurement of mental health status, and the General Health Questionnaire (GHQ), developed by the scholar, Goldberg, is one of the most representative instruments (12). The GHQ scale includes multiple versions, such as GHQ-60, GHQ-30, GHQ-20, GHQ-18, and GHQ-12. Among these, the GHQ-12 has become one of the most important tools for screening mental health status due to its simplicity and ease of operation (12, 13). It contains 12 items (six positive items and six negative items separately) (14), and is most commonly scored by 0-1-2-3 or 0-0-1-1 (15). Preliminary studies have found that the GHQ-12 has been translated into many versions and is widely used in different regions, such as Spain, Iran, Australia, India, and Sweden (16–20). It was also adopted to assess the mental health status of Chinese civil servants, adolescents, and university students (21–23).

Nevertheless, the reliability and validity of the GHQ-12 have not been clarified in Chinese healthcare workers, especially among dental healthcare workers. To address this issue, the purpose of this study was to evaluate the reliability and validity of the GHQ-12 and provide an effective tool for rapid screening of the mental health status of Chinese dental healthcare workers after the COVID-19 outbreak.

## Materials and Methods

### Participants

The data of this study were derived from the first occupational survey of dental healthcare workers in China conducted by the Chongqing Stomatological Association from February 2021 to March 2021. Based on the hospitals with a stomatological department in China, the Chongqing Stomatological Association used a convenience sampling method to send questionnaires by e-mail. Specifically, the staff of Chongqing Stomatological Association contacted the director in charge of the stomatological hospitals, the stomatological departments of the general hospitals, the dental clinics and invited them to participate in this research. Once the director agreed to participate in this study, the director invited the corresponding hospital/department healthcare workers to participate.

### Research Instruments

Each participant was provided with a self-administrated questionnaire consisting of two parts. Part one was the basic characteristics of dental healthcare workers, including gender, age, academic degree obtained, technician, monthly income, working years, hours worked per week, relationship status, having children or not, the number of patients treated per day, whether tube bed, hospital type, job type, major type, commuting time, and whether they undertaking teaching tasks. Part two was the GHQ-12. The scale reflects the mental health status of the respondents through 12 self-assessment items. Each item includes four options (A, B, C, and D), and the bimodal scoring method (0-0-1-1) was adopted. Specifically, If A or B was selected, the value equates to 0; if C or D was selected, the value equates to 1. The detection rate of mental health was considered to be positive (such as the feeling of depression and anxiety, insomnia, or suicidality) when the cumulative score ≥ four (24–26).

### Quality Control

To ensure the accuracy of the data, we evaluated the quality of all questionnaires. Criteria one: if the same answer was chosen for the whole questionnaire items, the questionnaire was considered invalid. Criteria two: we also checked the questionnaires from the same hospital. If two or more consecutive questionnaires had completely the same answers, only one questionnaire was included, and the other identical questionnaires were defined as invalid.

### Statistical Analysis

Data were entered and checked through Excel 2013, and all statistical analyses were conducted using SPSS 21.0 (IBM Corp., Armonk, NY) and AMOS 21.0. The mean and standard deviation (SD) was calculated for quantitative data, and the percentage was calculated for qualitative data. The scale reliability was assessed by Cronbach's α coefficient, Guttman Split-Half coefficient, and a coefficient >0.70 was considered acceptable (27). In addition, the Spearman correlation coefficient was used as an index to judge the reliability, and a value >0.30 was considered acceptable (28, 29). The higher the coefficient, the stronger the correlation between items. The scale construct validity was assessed by exploratory factor analysis (EFA) and confirmatory factor analysis (CFA). In this study, three-fourths of participants were assigned to EFA, and one-fourths were assigned to CFA. Specific indicators were used to evaluate the model fit. For EFA analysis, a Kaiser-Meyer-Olkin (KMO) coefficient >0.70, engine value >1.0 (30, 31) cumulative variance contribution rate >50%, and factor load >0.30 were recommended (32, 33). For CFA analysis, the Root Mean Square Error of Approximation (RMSEA) should be <0.10 (better if below 0.05); otherwise, the model should be rejected (33, 34). Comparative Fit Index (CFI), Normative Fit Index (NFI), Incremental Fit Index (IFI), Goodness of Fit Index (GFI) should be >0.90 (better if above 0.95) to indicate a proper fit (35–37). χ^2^/d*f* was extremely sensitive to the sample size and was not a suitable indicator for model fit for sample size above 200 (38, 39). Therefore, χ^2^/d*f* was not selected as an evaluation indicator in this study. A *P*-value < 0.05 was considered to be statistically significant.

## Results

### Demographic Characteristics

By the end of March 2021, a total of 3,128 questionnaires from 11 provinces in China were collected (Supplementary Table 1). There were 3,020 valid questionnaires (the effective rate was 96.55%) and 108 invalid questionnaires. Among these, 99 questionnaires were excluded by the exclusion criteria one, 9 questionnaires were excluded by the exclusion criteria two. For the respondents, 76.1% were female, 54.9% held a bachelor's degree, and 65.7% were 20–35 years old. 58.4% were junior technicians, and 45.4% had a monthly income between 5,000 and 10,000 RMB. Two-thirds worked <10 years, while 71.9% worked <45 h per week. 67.0% were married, 58.6% had children. 42.5% treated 10–20 patients per day. 88.6% tubed bed. 65.4% worked in a dental specialty hospital. 61.4% were doctors, and 44.1% worked in the general department. 40.9% commuted between 15 and 30 min, while two-thirds did not undertake teaching tasks. The demography characteristics are summarized in Table 1.

**Table 1:** Demography characteristics of the participants.

**Items**	***N* (%)**	**Items**	***N* (%)**
Gender		Have children	
Male	721 (23.9)	No	1,249 (41.4)
Female	2,299 (76.1)	Yes	1,771 (58.6)
Academic degree obtained		Treated patients per day	
Doctor's degree	137 (4.5)	<10	940 (31.1)
Master's degree	740 (24.5)	10–20	1,285 (42.5)
Bachelor's degree	1,659 (54.9)	20–30	415 (13.7)
College's degree or below	484 (16.0)	>30	380 (12.6)
Age, years		Whether tube bed	
20–35	1,984 (65.7)	No	2,675 (88.6)
35–50	862 (28.5)	Yes	345 (11.4)
>50	174 (5.8)	Hospital type	
Technician		Dental specialist hospital	1,975 (65.4)
Junior	1,763 (58.4)	General hospital	881 (29.2)
Intermediate	885 (29.3)	Private hospital	164 (5.4)
Senior	372 (12.3)	Job type	
Monthly income, RMB		Doctor	1,855 (61.4)
<5,000	842 (27.9)	Nurse	1,165 (38.6)
5,000–10,000	1,372 (45.4)	Major type	
10,000–15,000	415 (13.7)	General	1,331 (44.1)
>15,000	391 (12.9)	Internal medicine	662 (21.9)
Working years		Maxillofacial surgery	339 (11.2)
<10	2,009 (66.5)	Prosthodontics	269 (8.9)
10–20	636 (21.1)	Implant	114 (3.8)
>20	375 (12.4)	Orthodontics	305 (10.1)
Hours worked per week		Commuting time, minutes	
<45	2,172 (71.9)	<15	544 (18.0)
45–55	639 (21.2)	15–30	1,235 (40.9)
>55	209 (6.9)	30–45	615 (20.4)
Relationship status		45–60	397 (13.1)
Single	565 (18.8)	>60	229 (7.6)
Partnered	359 (11.9)	Undertake teaching tasks	
Married	2,022 (67.0)	Yes	1,010 (33.4)
Divorced or widowed	72 (2.4)	No	2,010 (66.6)

*N, the number of participants, %, the proportion of the participants*.

### GHQ-12 Characteristics

The positive rate of mental health status was 23.80% (719/3,020). The quartile of the GHQ-12 total score was 2 (0, 5) points, which was lower than the positive detection rate threshold. Among all items, item 12 had the lowest frequency of answering 1. In contrast, more than a quarter of the participants responded positively (answered 1) among item 1 and item 2, indicating that the positive findings in mental health status were mainly caused by “Lost much sleep” and “Under stress”, as shown in Table 2.

**Table 2:** Item respondents of GHQ-12 items (*n* = 3,020).

**Items**	**Item positive/respond rate (%)**			
	**A**	**B**	**C**	**D**
1.Lost much sleep	1,025 (33.9)	1,161 (38.4)	707 (23.4)	127 (4.2)
2.Under stress	434 (14.4)	1,450 (48.0)	933 (30.9)	203 (6.7)
3.Able to concentrate	237 (7.8)	2,183 (72.3)	534 (17.7)	66 (2.2)
4.Playing a useful part	440 (14.6)	2,282 (75.6)	249 (8.2)	49 (1.6)
5.Face up to problems	357 (11.8)	2,307 (76.4)	312 (10.3)	44 (1.5)
6.Capable of making decisions	357 (11.8)	2,329 (77.1)	300 (9.9)	34 (1.1)
7.Could not overcome difficulties	804 (26.6)	1,795 (59.4)	381 (12.6)	40 (1.3)
8.Feeling reasonably happy	311 (10.3)	2,080 (68.9)	519 (17.2)	110 (3.6)
9.Enjoy your day-to-day activities	276 (9.1)	2,090 (69.2)	539 (17.8)	115 (3.8)
10.Feeling unhappy and depressed	618 (20.5)	1,709 (56.6)	584 (19.3)	109 (3.6)
11.Losing confidence	1,199 (39.7)	1,413 (46.8)	359 (11.9)	49 (1.6)
12.Thinking of self as worthless	1,587 (52.5)	1,159 (38.4)	236 (7.8)	38 (1.3)

### Reliability and Correlations Analysis

The Cronbach's α coefficient of the whole GHQ-12 was 0.892. After excluding individual items, the Cronbach's α coefficient of the total scale ranged from 0.877 to 0.888, all of which were lower than the overall scale's 0.892 (Table 3). The Spearman-Brown coefficient of the half-fold reliability of the scale was 0.843, indicating that the scale's reliability was acceptable.

**Table 3:** Cronbach's α and correlations for GHQ-12 scale (*n* = 3,020).

**Items**	**Cronbach's α after the item deleted**	**Correlation coefficient with total score**
1.Lost much sleep	0.887	0.680
2.Under stress	0.888	0.762
3.Able to concentrate	0.886	0.596
4.Playing a useful part	0.887	0.467
5.Face up to problems	0.881	0.539
6.Capable of making decisions	0.885	0.488
7.Could not overcome difficulties	0.885	0.519
8.Feeling reasonably happy	0.878	0.676
9.Enjoy your day-to-day activities	0.881	0.658
10.Feeling unhappy and depressed	0.877	0.701
11.Losing confidence	0.882	0.552
12.Thinking of self as worthless	0.886	0.465

Spearman correlation analysis showed that the correlation coefficient between the item score and total scores was 0.465–0.762 (*P*<0.05). Item 2 showed the highest correlation with the total score, and item 12 showed the lowest correlation with the total score, which was higher than the criterion of 0.30 (Table 3). The correlation ranged from 0.264 to 0.665 among the inter-item scores (Supplementary Table 2). Item 2 showed the lowest correlation with item 12, and item 8 had the highest correlation with item 10. The correlation coefficient between the items-total score was higher than the inter-items, indicating the reliability of this scale was acceptable.

### Exploratory Factor Analysis

KMO and Bartlett sphericity tests were performed on 12 items of the scale, and the KMO coefficient was 0.924, χ^2 =^ 11,642.038, d*f* =66, and *P*-value <0.001, indicating that it was suitable for factor analysis. The maximum variance method was used for principal component analysis, and two common factors were extracted with eigenvalues >1, as shown in Supplementary Figure 1. Common factor 1 contains 6 items, namely items 1, 2, 3, 8, 9, 10, with a variance contribution rate of 31.999%. Common factor 2 contains 6 items, namely item 4, 5, 6, 7, 11, and 12, with a variance contribution rate of 24.767%. The cumulative variance contribution rate of the two-factor model showed a total variance of 56.766%, reaching the acceptable standard of more than 50% (32, 33) (Table 4). In addition, the factor load of items ranged from 0.564 to 0.818, which was higher than the recommended value of 0.30.

**Table 4:** Factor load of the exploratory factor analysis for GHQ-12 (*n* = 2,265).

**Items**	**Common factor 1**	**Common factor 2**
1.Lost much sleep	0.133	0.801
2.Under stress	0.115	0.818
3.Able to concentrate	0.325	0.593
4.Playing a useful part	0.708	0.137
5.Face up to problems	0.729	0.284
6.Capable of making decisions	0.690	0.223
7.Could not overcome difficulties	0.606	0.304
8.Feeling reasonably happy	0.525	0.564
9.Enjoy your day-to-day activities	0.463	0.573
10.Feeling unhappy and depressed	0.527	0.572
11.Losing confidence	0.721	0.267
12.Thinking of self as worthless	0.735	0.153
Variance contribution rate	31.999%	24.767%
Cumulative variance contribution rate	56.766%	56.766%

### Confirmatory Factor Analysis

**Figure 1:**
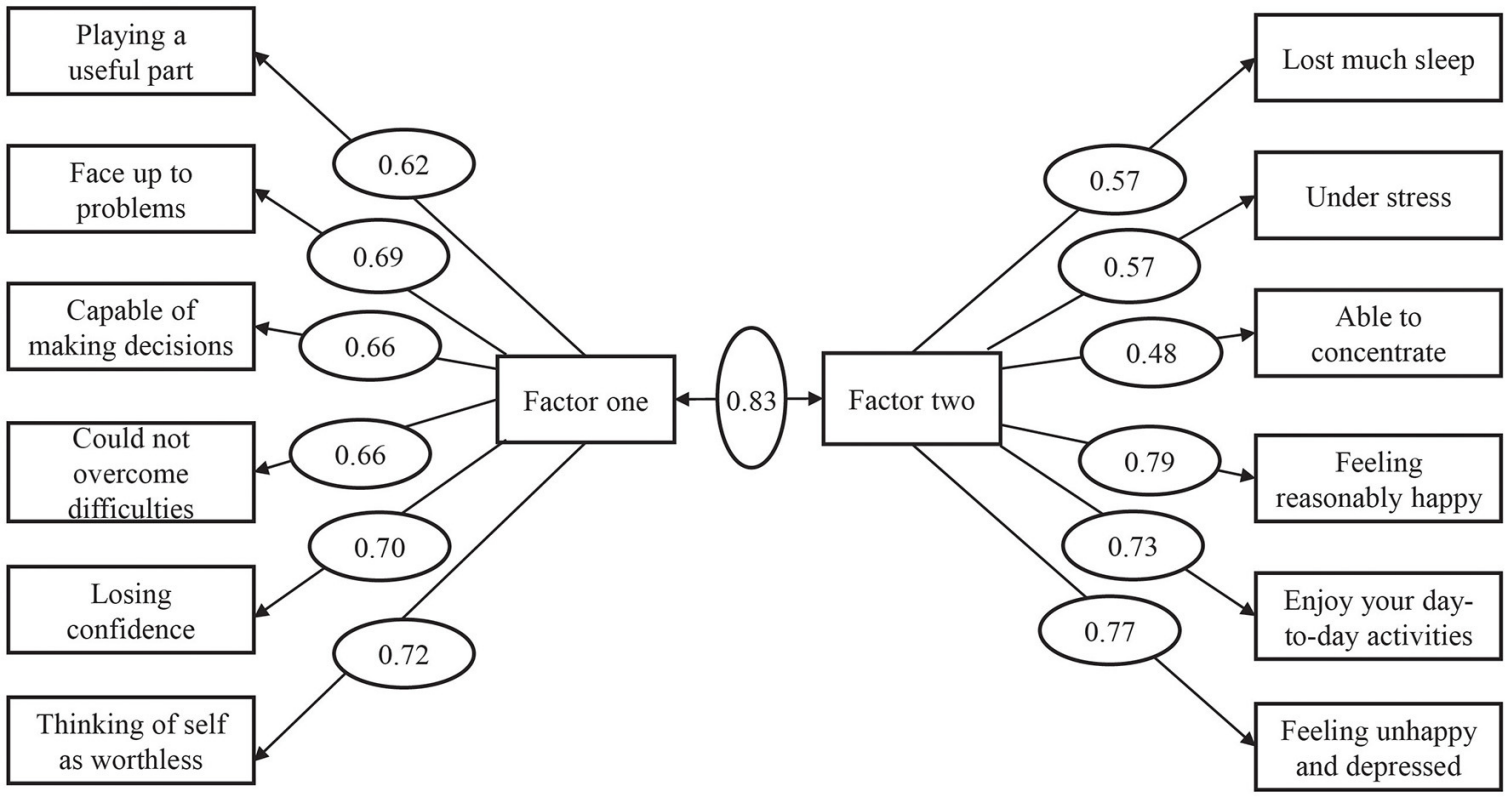
Standard factor load of two common factor model.

The χ^2^/d*f* value of the original model was 5.795, with an RMSEA value of 0.08. The values of NFI, CFI, IFI, TLI, GFI were 0.914, 0.928, 0.928, 0.910, 0.933, respectively (Table 5). The correlation coefficient between common factor one and common factor two was 0.83, and the factor load on the specified factors of each item of the AMOS path ranged from 0.48 to 0.79 (Figure 1). All of the indexes were greater than the recommended value, indicating the validity of the two-factor model of GHQ-12 was acceptable. Based on the previous studies that explored the factor structure of GHQ-12 (14, 39–44), we also validated the previous factor model using our CFA sample size. All of the models produced relatively similar results. The RMSEA was more than 0.090, and NFI, as well as TLI, were >0.90 in all models. CFI, IFI and GFI were less than the recommended value of 0.90 in most models. The fit index of the CFA model is displayed in Table 5.

**Table 5:** Index of the confirmatory factor analysis for GHQ-12 (*n* = 755).

**Index**	**Unidimensional**	**Andrich**	**Schmitz**	**Politi**	**Graetz**	**Farrell**	**Daradkeh**	**Original**
RMSEA	0.101	0.101	0.105	0.099	0.095	0.103	0.103	0.080
χ^2^/d*f*	8.666	8.662	9.344	8.406	7.760	8.961	8.942	5.795
NFI	0.869	0.872	0.876	0.880	0.890	0.872	0.873	0.914
CFI	0.882	0.885	0.887	0.892	0.902	0.885	0.885	0.928
IFI	0.883	0.885	0.888	0.893	0.902	0.885	0.885	0.928
TLI	0.856	0.856	0.856	0.862	0.873	0.851	0.851	0.910
GFI	0.896	0.897	0.905	0.915	0.911	0.899	0.899	0.933

*RMSEA, root mean squared error of approximation; NFI, normed fit index; CFI, comparative fit index; IFI, incremental fit index; TLI, tucker-lewis index; GFI: goodness-of-fit index*.

## Discussion

GHQ-12 is one of the most widespread scales to measure the mental health status of well-being, and has been translated to many versions worldwide (16–20). Although GHQ-12 has been frequently used in China, a few studies from China also tested the reliability and validity of GHQ-12 in civil servants, university students, and adolescents (21–23). There is no study to test the factor structure of GHQ-12 in Chinese healthcare workers. As we know, healthcare workers are at high-risk developing mental health problems due to their professional attributes, such as the huge workload, caregiver burden, high levels of job stress and low levels of job satisfaction (25, 45). Furthermore, mental health problems can lead to burnout, which will increase the risk of patient safety and decrease the healthcare quality (46, 47). Particularly, for dental healthcare workers, it is worse because of the COVID-19 outbreak and the working environment is prone to aerosol. To our knowledge, this is the first study evaluating the reliability and validity of GHQ-12, with the hope of providing an instrument for the quick assessment of the mental health status of dental healthcare workers in China.

In the current study, we gathered 3,020 valid electronic questionnaires of dental healthcare workers from 11 provinces in China. We believe this is the first large-scale use of GHQ-12 to assess the mental health status among dental healthcare workers in China. The positive rate of the mental health status of GHQ-12 was 23.8%, which was close to the healthcare workers in Nigeria (23.4%) (48), lower than the Chinese neurologists (37.8%) (25), healthcare workers in Japan (65.6%) (49), doctors in emergency medicine, anesthesia and intensive care medicine in the UK and Ireland (44.2%) (50) and obstetrics healthcare providers in Italy (51.1%) (51). It illustrated that the positive rate of mental health status of dental healthcare workers was relatively low, which could be attributed to the effective containment of COVID-19. However, nearly a quarter of participants had a positive detection of mental health status, which suggested that dental healthcare workers are still at high risk of mental health status.

In this study, Cronbach's α, Spearman-Brown coefficient, Spearman correlation analysis, EFA and CFA measurement were evaluated. On the basis of the reliability, the Cronbach's α coefficient of the total scale was 0.892. After removing individual items, the Cronbach's α coefficient of the rest of the scale was lower than 0.892 but >0.70, which means that deleting any item may reduce the reliability of the total scale. Furthermore, it also illustrates that individual items were important and cannot be deleted from the total scale, confirming previous studies' results (21). The Spearman-Brown coefficient of the total scale was 0.843, which was higher than the recommended value. In addition, the Spearman correlation coefficient of the item-total score was higher than the inter-item correlation coefficient, indicating that the reliability of the scale was appropriate, which was consistent with the published study (21).

To indicate the validity, EFA and CFA were performed to extract the common factor and verify the model separately. EFA extracted two common factors with engine-value >1.0 from GHQ-12, and the factor load was relatively high, ranging from 0.564 to 0.818. The results showed that the cumulative variance contribution rate of the two-factor model to the total variation of the GHQ-12 scale was 56.766%, which was higher than that of civil servants in China (50.22%), adolescents in China (53.27%) and the recommended value (50.0%) (32, 33). Furthermore, the CFA results showed that the RSMEA of the two-factor model was less than the recommended value of 0.10, and the fitness index NFI, IFI, TLI, GFI were higher than the recommended value of 0.90. The results are similar to the previous studies (21, 52), which means our two-factor model could interpret the mental health status of dental healthcare workers in China.

Historical studies have proposed many classical dimensional models, including unidimensional, two-dimensional and three-dimensional models (14, 39–44). Andrich proposed a classical two-dimensional model with 12 items, including six positive items (items 1, 3, 4, 7, 8, 12) and six negative items (items 2, 5, 6, 9, 10, 11) (14). Graetz proposed a classical three-dimensional model with a total of 12 items, including social disorder (items 1, 3, 4, 7, 8, 12), anxiety and depression (items 2, 5, 6, 9), and lack of confidence (items 6, 9) (42). However, in our results, common factor 1 included items 1, 2, 3, 8, 9, 10, and common factor 2 included items 4, 5, 6, 7, 11, 12. The two-factor model obtained from our study was distinct from the above models, which might be caused by the regional difference and research population difference. In addition, we also conducted CFA analysis to validate the GHQ-12 based on the seven previous classical models using our sample, and these seven classical models yield relatively similar results. Compared with the above classical models, the results also showed the model performance was superior to the other seven classical models based on the CFA fit index. This demonstrates that the two-factor model we proposed has good structural validity, and is suitable for evaluating the mental health status of Chinese dental healthcare workers.

## Conclusions

Nearly a quarter of dental healthcare workers had mental health problems. The two-factor model of GHQ-12 we put forward had good reliability and validity among dental healthcare workers, which could be used as an instrument to screen the mental health status of dental healthcare workers in China.

## Limitations

There are also some limitations in our study: Firstly, this study adopted a convenience sampling method, so the data may be prone to selection bias. Secondly, this study did not set a gold standard for measuring the mental health status, and lacked an efficacy standard validity analysis. Thirdly, we also did not conduct the reset reliability analysis. Fourthly, the characterization of non-respondents was not available, therefore it cannot be compared with the valid respondents. Finally, the study carried out on the population of healthcare workers, so it may not applicable to the general or other professional populations. Due to the above limitations, more studies are needed to support our results.

## Data Availability Statement

The original contributions generated for this study are included in the article or supplementary material, further inquiries can be directed to the corresponding author.

## Ethics Statement

The front page of the questionnaire introduced the background and purpose of the survey, informed participants of their rights and potential risks, and informed them that the survey was voluntary and anonymous. So, the informed consent was presumed if the participants returned the questionnaires. The questionnaire and study protocol were reviewed and approved by the ethics committee of the Stomatological Hospital of Chongqing Medical University.

## Author Contributions

XZ and XJ: proposed the concept and design. LYan, LYang, HL, and JW: collected the data. XZ and XJ: analyzed and interpreted the data, wrote the manuscript. HW, YL, and JP: drafted and edited the manuscript. XJ, PJ, and PX: provided the funding. PX and PJ: supervised the study. All authors reviewed and approved the manuscript prior to its submission, and the authors declare no competing interests.

## Funding

This work was supported by the Natural Science Foundation Project of China (Grant No. 81870775, 81500855) and the National Key R&D Program of China (Grant No. 2017YFA0505700).

## Conflict of Interest

The authors declare that the research was conducted in the absence of any commercial or financial relationships that could be construed as a potential conflict of interest.

## Publisher's Note

All claims expressed in this article are solely those of the authors and do not necessarily represent those of their affiliated organizations, or those of the publisher, the editors and the reviewers. Any product that may be evaluated in this article, or claim that may be made by its manufacturer, is not guaranteed or endorsed by the publisher.
