# Supplementary material for: Reliability and Validity of General Health Questionnaire-12 in Chinese Dental Healthcare Workers During the COVID-19 Pandemic
Source: Front Psychiatry. 2022 Jan 18;12:792838. doi: 10.3389/fpsyt.2021.792838 (PMC8805792; doi:10.3389/fpsyt.2021.792838)
Supplement: Supplementary file 1 [file Data_Sheet_1.docx]

| Supplementary Material  **Supplementary Table 1 \|** Number of participates of each province. | |
| --- | --- |
| **Province** | **Number of participants** |
| Chongqing | 1641 |
| Yunnan | 368 |
| Guizhou | 283 |
| Xinjiang | 231 |
| Sichuan | 203 |
| Neimeng | 124 |
| Ningxia | 103 |
| Gansu | 54 |
| Qinghai | 6 |
| Xizang | 6 |
| Shanxi | 1 |
| **Total** | **3020** |

**Supplementary Table 2 |** The spearman correlation coefficient between items and total score/items.

| Items | GHQ-1 | GHQ-2 | GHQ-3 | GHQ-4 | GHQ-5 | GHQ-6 | GHQ-7 | GHQ-8 | GHQ-9 | GHQ-10 | GHQ-11 | GHQ-12 |
| --- | --- | --- | --- | --- | --- | --- | --- | --- | --- | --- | --- | --- |
| GHQ-1 | 1 |  |  |  |  |  |  |  |  |  |  |  |
| GHQ-2 | 0.585^**^ | 1 |  |  |  |  |  |  |  |  |  |  |
| GHQ-3 | 0.432^**^ | 0.436^**^ | 1 |  |  |  |  |  |  |  |  |  |
| GHQ-4 | 0.289^**^ | 0.279^**^ | 0.339^**^ | 1 |  |  |  |  |  |  |  |  |
| GHQ-5 | 0.348^**^ | 0.336^**^ | 0.417^**^ | 0.521^**^ | 1 |  |  |  |  |  |  |  |
| GHQ-6 | 0.296^**^ | 0.304^**^ | 0.370^**^ | 0.441^**^ | 0.597^**^ | 1 |  |  |  |  |  |  |
| GHQ-7 | 0.315^**^ | 0.325^**^ | 0.338^**^ | 0.376^**^ | 0.472^**^ | 0.433^**^ | 1 |  |  |  |  |  |
| GHQ-8 | 0.415^**^ | 0.408^**^ | 0.416^**^ | 0.377^**^ | 0.505^**^ | 0.434^**^ | 0.456^**^ | 1 |  |  |  |  |
| GHQ-9 | 0.401^**^ | 0.409^**^ | 0.376^**^ | 0.337^**^ | 0.452^**^ | 0.405^**^ | 0.418^**^ | 0.615^**^ | 1 |  |  |  |
| GHQ-10 | 0.422^**^ | 0.433^**^ | 0.410^**^ | 0.371^**^ | 0.483^**^ | 0.415^**^ | 0.446^**^ | 0.665^**^ | 0.611^**^ | 1 |  |  |
| GHQ-11 | 0.321^**^ | 0.324^**^ | 0.347^**^ | 0.448^**^ | 0.477^**^ | 0.434^**^ | 0.442^**^ | 0.502^**^ | 0.467^**^ | 0.551^**^ | 1 |  |
| GHQ-12 | 0.304^**^ | 0.264^**^ | 0.350^**^ | 0.475^**^ | 0.459^**^ | 0.406^**^ | 0.416^**^ | 0.395^**^ | 0.369^**^ | 0.432^**^ | 0.598^**^ | 1 |
| Total score | 0.680^**^ | 0.762^**^ | 0.596^**^ | 0.467^**^ | 0.539^**^ | 0.488^**^ | 0.519^**^ | 0.676^**^ | 0.658^**^ | 0.701^**^ | 0.552^**^ | 0.465^**^ |

**p<0.05; **p<0.01.*


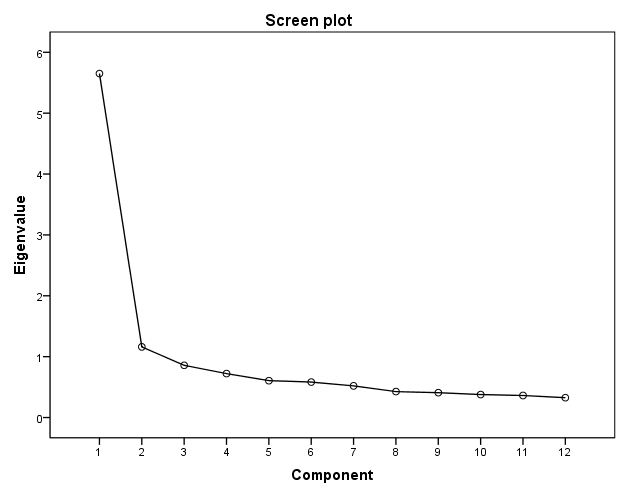


**Supplementary** **Figure 1 |** Screen plot of the exploratory factor analysis.
